# Development of consensus-based considerations for use of adult proxy reporting: an ISOQOL task force initiative

**DOI:** 10.1186/s41687-023-00588-6

**Published:** 2023-06-02

**Authors:** Brittany Lapin, Matthew L. Cohen, Nadia Corsini, Alyssa Lanzi, Sarah C. Smith, Antonia V. Bennett, Nancy Mayo, Rebecca Mercieca-Bebber, Sandra A. Mitchell, Claudia Rutherford, Jessica Roydhouse

**Affiliations:** 1 Department of Quantitative Health Sciences, Lerner Research Institute, Cleveland Clinic, 9500 Euclid Avenue, JJN3, Cleveland, OH 44195 USA; 2 Center for Outcomes Research and Evaluation, Neurological Institute, Cleveland Clinic, Cleveland, OH USA; 3 Department of Communication Sciences and Disorders, University of Delaware, Newark, DE USA; 4 Clinical and Health Sciences, Rosemary Bryant AO Research Centre, University of South Australia, Adelaide, SA Australia; 5 Department of Health Services Research and Policy, London School of Hygiene and Tropical Medicine, London, UK; 6 Lineberger Comprehensive Cancer Center, University of North Carolina at Chapel Hill, Chapel Hill, NC USA; 7 School of Physical and Occupational Therapy, Divisions of Clinical Epidemiology, Geriatrics, Experimental Medicine, Department of Medicine, Center for Outcomes Research and Evaluation (CORE) McGill University Health Centre (MUHC)-Research Institute, McGill University, Montreal, Canada; 8 NHMRC Clinical Trials Centre, The University of Sydney, Sydney, NSW Australia; 9 Outcomes Research Branch, Healthcare Delivery Program, National Cancer Institute, Bethesda, MD USA; 10 Faculty of Medicine and Health, The University of Sydney Susan Wakil School of Nursing and Midwifery, Cancer Care Research Unit (CCRU), The University of Sydney, Sydney, NSW Australia; 11 Faculty of Science, School of Psychology, Sydney Quality of Life Office, The University of Sydney, Sydney, NSW Australia; 12 Menzies Institute for Medical Research, University of Tasmania, Hobart, TAS Australia; 13 Department of Health Services, Policy and Practice, Brown University School of Public Health, Providence, RI USA

**Keywords:** Proxy-reported outcomes, Proxy reporting, Observer reporting, Targeted review

## Abstract

**Aims:**

Many large-scale population-based surveys, research studies, and clinical care allow for inclusion of proxy reporting as a strategy to collect outcomes when patients are unavailable or unable to provide reliable self-report. Prior work identified an absence of methodological guidelines regarding proxy reporting in adult populations, including who can serve as a proxy, and considerations for data collection, analysis, and reporting. The primary objective of this work by the ISOQOL Proxy Task Force was to review documents and clinical outcome assessment measures with respect to proxy reporting and to develop, through consensus, considerations for proxy reporting.

**Methods:**

We assembled an international group with clinically relevant and/or methodological expertise on proxy use in adult populations. We conducted a targeted review of documentation based on regulatory, non-regulatory, professional society, and individual measure sources. Using a standardized collection form, proxy-related information was extracted from each source including definitions of a proxy, characteristics of a proxy, domains addressable or addressed by a proxy, and observer-reporting.

**Results:**

The definition of proxy was inconsistent across 39 sources, except regulatory documents which defined a proxy as a person other than the patient who reports on an outcome as if she/he were the patient. While proxy report was discouraged in regulatory documentation, it was acknowledged there were instances where self-report was impossible. Many documentation sources indicated proxies would be well-justified in certain contexts, but did not indicate who could act as a proxy, when proxies could be used, what domains of patient health they could report on, or how data should be reported. Observer-reported outcomes were typically defined as those based on observed behaviors, however there was not a consistent differentiation between proxy and observer reporting. Based on information extracted from these resources, we developed a checklist of considerations when including proxy-reported measures or using proxies in study design, data collection, analysis, interpretation and reporting of proxy reported data.

**Conclusion:**

Our targeted review highlights a lack of clarity in capturing, interpreting and reporting data from proxies in adult populations. We provide a checklist of considerations to assist researchers and clinicians with including proxies in research studies and clinical care. Lastly, our review identified areas where further guidance and future research are necessary.

**Supplementary Information:**

The online version contains supplementary material available at 10.1186/s41687-023-00588-6.

## Background

Clinical outcome assessments (COAs) play an important role in many clinical contexts. The umbrella term COA covers different types of assessments, including patient-reported outcomes (PROs), clinician-reported outcomes (ClinROs) and observer-reported outcomes (ObsROs) [1]. PROs, a subset of COAs, are used in many clinical contexts to provide information by self-report about patient health, function and health-related quality of life (QoL). Regulatory agencies such as the European Medicines Agency (EMA) [2] and US Food and Drug Administration (FDA) [3] have encouraged the inclusion of patient-reported data in the drug development process, and there is also recognition of the importance of PRO data in registries for tracking patients’ perspectives of their health [4]. Critically, in many clinical contexts patients’ may be unable to provide health information owing to illness, older age, or cognitive impairment. Proxies (e.g., caregivers), are often used to report on these patient-centric outcomes in these situations.

Although there has been research on proxy reporting, a substantial challenge is the heterogeneity and lack of clarity and consistency regarding the terminology used to describe and define proxies [5–7]. For example, in some measures used in palliative care, one of the settings in which proxies are an important consideration, clinicians are considered proxies [8]. However, as noted previously, the FDA considers clinician-reported outcomes to be a specific type of COA [9]; furthermore, ObsROs are likewise a specific type of COA. Proxy-reported outcomes (ProxRO) have been considered a type of ObsRO or a separate COA. Regulatory definitions of proxies typically distinguish ProxROs from ObsROs by the presence of a perspective, i.e. the proxy reports as if they were the patient [2, 3]. However, the proxy’s perspective is rarely recorded in many applications [5], and the literature describes more than one type of perspective for proxies [10]. This inconsistency and lack of clarity regarding definitions poses substantial challenges for interpreting studies across different clinical areas, and for researchers considering different measures for their studies.

In recognition of the evidence gap and the absence of consensus guidelines regarding the use of proxies in research with adult populations, the International Society for Quality of Life (ISOQOL) Proxy Task Force was formed. The Task Force recently completed a large-scale review that described the use of proxies and proxy-reported measures in adult health studies [7]. The review highlighted several areas in which further work is needed, including but not limited to the lack of clarity regarding who can be a proxy (e.g., based on relationship type and knowledge of the patient), how to differentiate proxy- and observer-reported measures, and standards of development for proxy-reported measures.

To address these issues and provide consensus recommendations for the inclusion of proxy reporting, the ISOQOL Proxy Task Force reviewed the literature and guidelines available from professional societies, regulators and other organizations to identify considerations for the use of proxies and proxy-reported measures. The Task Force’s primary objective in this targeted review was to evaluate the available definitions and recommendations regarding the use of proxy reporting for adults and to develop, by consensus, a checklist of considerations for the use of proxies in adult health studies.

## Methods

### Targeted review

Between September 2021 and March 2022, a targeted review was conducted to evaluate the definitions of proxies and proxy measures in different clinical contexts and from different bodies (i.e., regulatory agencies, measure developers, etc.). A targeted review was considered appropriate for this study due to its focus on developing considerations. Many measures were identified based on our prior review [7], however most were developed for single-use and were not applicable in the development of best practices. We therefore chose to focus on key guidance documents and more commonly used measures in areas where proxy use was expected to be highly relevant.

The Task Force members for this study included an international group of experts on proxy reporting. Additional experts were recruited for this study who work in clinical areas with frequent proxy use including stroke, cancer, rehabilitation, dementia, and palliative care. The targeted review included evidence from a range of sources including documents published by governmental and regulatory agencies, such as the FDA [3], good practice reports and standards from relevant professional societies and organizations, such as ISOQOL [11], and standard measure sets and measures for conditions with frequent proxy use (see Table 1). The study team met to discuss and finalize screening decisions for inclusion of documents. When selecting individual measures, we focused on commonly used measures in contexts known to require proxies, or to be particularly well-known in specific clinical areas such as dementia, as well as guidelines relating to the administration and interpretation of specific PROs. These determinations were based on Task Force member expertise. The EQ-5D was included as a generic measure that is widely used in conditions where proxy use is common, such as aging populations, stroke, and traumatic brain injury. Study team members sourced the documents they were most familiar with or that aligned with their known content areas.

**Table 1:** Summary of targeted review documents relevant to proxy reporting

Type of resource	Resource	Use of proxy-reporting addressed	When to employ proxies	Proxy relationship /characteristics	Proxy perspective	Domains evaluated by proxy
Regulatory or scientific agencies	FDA PRO guidance [3]	Yes	Discouraged, particularly for symptoms known only by the patient	Someone other than the patient	Proxy-patient perspective	Proxy reports as patient; observer reports on something observable and may also provide judgment
	FDA patient-focused drug development (PFDD) guidance [12, 13]	Yes	Discouraged, may not be reflective of patient’s feelings; For patients who are unable to self-report, eliciting behaviors caregivers observe can help avoid proxy reporting	Caregiver; someone other than the patient or health professional; ObsRO are reported by parent, caregiver, or someone who observed the patient in daily life	Proxy-patient perspective	None; ObsROs can report on observations but do not include judgment
	FDA PFDD guidance appendices [14]	Yes	Discouraged, but ObsRO recommended in instances where it is impossible to have self-report	Someone who is not the patient; ObsRO are reported by parent, caregiver, or someone who observed the patient in daily life	Proxy-patient perspective	Discouraged for concepts only known by the patient (e.g. symptoms)
	EMA Appendix 2 (use of PROs in oncology) [2]	Yes	Should be avoided, unless it is the only means of obtaining lost information. Use when patients cannot contribute (e.g. cognitive impairment, severe ill health)	Caregivers (non-clinical)	Proxy-patient perspective	Discouraged; ObsROs can report on observations but do not include judgement
	CMS measures management system supplement on PROs [15]	Yes	Measure developer needs to consider whether to allow them	–	–	–
	AHRQ user’s guide on registries for evaluating patient outcomes [4]	Yes	If the only available measure, for patients not physically or cognitively able to provide direct assessment	Close family or caregivers	Proxy-patient perspective	–
Non regulatory	PCORI standards for PROs [16]	Yes	When patients are unable to self-report	Caregivers	–	Observable events or behaviors
	OECD strengthening health system [17]	Yes	For non-response in certain disease groups, use proxy/observer	Caregivers	–	References DEMQOL and DEMQOL-Proxy
	NQF PRO-PM 2013 [18]	Yes	–	–	–	–
	NQF PRO-PM roadmap 2020 [19]	Yes	Patients with severe physical or cognitive impairments	Caregivers, family members, or other people	–	–
	Development of a model for a National Dementia Registry [20]	Yes	In advanced dementia cases, suggest a proxy version of QOL-AD	Primary caregiver	–	References QOL-AD
Professional society	ISOQOL minimum standards [21]	No	–	–	–	–
	ISOQOL’s User Guide to Implementing PROs in Clinical Practice [11]	Yes	Patients requiring assistance (incapacities, mentally or cognitively limited)	Significant others, caregivers, physicians	–	–
	ISOQOL Dictionary [22]	No	Observers cannot report on how another person feels but only on what they are observed to do	–	–	–
	ISPOR emerging good practices task force: conceptual foundation [23]	No	Discusses observer rater to contrast with clinical professionals for performing ObsRO (vs ClinRO) assessments	Caregiver, parent, spouse, companion (not clinician)	–	–
	ISPOR emerging good practices task force: rare disease [24]	Yes	ObsRO measure must be based on observations and are not proxy measures	–	–	–
	Montreal accord on PROs [25]	Yes	–	Proxy is a special kind of observer with a “shared experience” [with the patient] that enables them to “report on the outcome”	–	Symptoms, physical impairments, behavior, and function
Recommendations/guidelines	SPIRIT-PRO [26, 27]	Yes	Per EMA/FDA, discourage use of proxies unless they provide the only means of obtaining information that may be lost	Someone other than the trial participant	Proxy-patient perspective	–
	CONSORT-PRO [28]	Yes	In some instances it may not be possible for the PRO to be completed directly by the patient	Caregiver, clinician	Proxy-patient perspective	–
	MORECare consensus on outcome measurement in palliative care [29]	Yes	Deterioration or unstable symptoms with physical and/or mental debility	Clinicians or non-clinicians; Capture factors that may influence proxy raters	–	–
Standard measure sets	ICHOM Oncology [30]	No	–	–	–	–
	ICHOM Stroke [31]	Yes	For patients unable to respond	Proxy, clinician, or abstracted from medical records	–	Proxy should make a judgement on observable domains only
	COMET [32]	No	–	–	–	–
Measure developers	EORTC Measures Manual (cancer) [33]	Yes	Currently exploring the value of proxy assessment, which may be useful in patients with major cognitive problems	–	–	–
	Guidelines for Assessing QoL in EORTC Clinical Trials [34]	Yes	When difficult or impossible for patients to rate their own QoL (e.g. cognitive impaired or terminally ill)	A family member (e.g. partner or parent) or “care taker” (e.g. physician or nurse)	–	–
	FACIT [35]	Yes	Suggest a sequence: (1) self-report; (2) self-report with assistance from an objective person; (3) self-report with assistance from a familiar person; (4) proxy report	A familiar person (e.g. friend or family member)	–	–
	PROMIS [36]	Yes	Patients unable to answer on their own, such as people in the early stage of dementia, cognitive or communication deficits, and severe disease burden	Someone else	–	–
Individual measures (for conditions with expected high use of proxy report)	QOLAS (dementia) [37]	Yes	Measure includes an assessment from caregivers	Caregivers	Proxy-proxy perspective	Physical, psychological, social/family, daily activity, cognitive
	QOL-D (dementia) [38]	Yes	–	Members of nursing staff or family well acquainted with the patient	–	Positive and negative affect, restlessness, attachment with others, spontaneity and activity
	DEMQOL and DEMQOL Proxy (dementia) [39, 40]	Yes	Measure includes an assessment from caregiver; In people with severe dementia, only DEMQOL-Proxy should be used due to high levels of missing data for DEMQOL	Family caregiver	Proxy-patient perspective	Daily activities, health and well-being, cognitive function, social relationships, self-concept (all subjective)
	Cornell-Brown scale for quality of life (dementia) [41]	Yes	Measure includes an assessment from caregiver; Professional completes the scale after interviewing patient and caregiver	Caregiver with daily contact with the patient	–	Mood ratings, ideational disturbance, physical signs, cyclic functions
	QOL-AD (Alzheimer’s, dementia) [42, 43]	Yes	Proxy perspective can be obtained for any participant, but those with MMSE ≤ 10 may be unable to answer	Actively involved caregiver who lived with patient or spent every day with them	Proxy-patient perspective	Perceived quality of life
	ADRQL (Alzheimer’s) [44, 45]	Yes	Measure includes an assessment from caregiver	Close family members or professional caregivers	–	Social interaction, awareness of self, feelings and mood, enjoyment of activities, response to surroundings
	MD Anderson Symptom Inventory user guide (cancer) [46]	No	–	–	–	–
	EORTC QoL Questionnaires (cancer) [47]	No	–	–	–	–
	Neuro-QoL (neurological disorders) [48]	Yes	Patients unable to answer on their own, such as people in the early stage of dementia, cognitive or communication deficits, and severe disease burden	Caregiver proxy responders; Someone else	Proxy-patient perspective	–
	ASCQ-Me (sickle cell) [49]	No	–	–	–	–
	Palliative Care Research Cooperative Measurement Tool Library [50]	Yes	–	Family caregiver	–	Refer to individual PRO user manuals for specific details
	EQ-5D [51]	Yes	Proxy versions developed for cases where patients are mentally/physically incapable of self-reporting	A caregiver who knows the patient well (e.g. parent, physician, nurse)	Proxy version 1 is Proxy-proxy perspective; Proxy version 2 is Proxy-patient perspective	Mobility; self-care; usual activities; pain/discomfort; anxiety/depression; health today

*ObsRO* observer-reported outcome, *ClinRO* clinician-reported outcome, *QOL* quality of life, *MMSE* mini-mental state exam for cognitive function among the elderly, *PRO* patient-reported outcome

A data extraction form was created by the Task Force co-chairs (BL, JR) (Additional file 1: Appendix 1), with the following information extracted from each resource to facilitate synthesis: (1) definition of proxy, including whether the relationship of the proxy was specified, as well as any other characteristics; (2) definition of proxy report, including whether a perspective, judgment or type of outcome was specified; (3) definition of observer, including whether the relationship or characteristics were specified; and (4) observer report, including whether a judgment or type of outcome was specified. The extracted information from the individual forms was entered into a spreadsheet for ease of comparison. For simplicity, if proxies were described as either carers or caregivers, we used the term caregivers for consistency. Prior work has demonstrated that the definition of which ‘caregiver’ can act as a proxy includes both paid and unpaid caregivers and may sometimes include clinicians [7]. Thus, for this study we did not impose a restriction or firm definition on the term.

### Consensus development of considerations for proxy reporting

Multiple meetings were held by the study team to synthesize the findings, including discussion of the commonalities and differences among document sources. The synthesized findings were summarized by topic, with a broad overview presented in Table 1. A checklist of considerations for the use of proxies and proxy-reported measures was developed and structured under thematic headings related to the stage of consideration: study design, data collection methods, analytic methods, outcomes interpretation, and reporting recommendations. The checklist was initially developed by the Task Force co-chairs, and reviewed and revised by all authors.

Ethics approval was not required for this study.

## Results

Information was extracted from 39 sources spanning regulatory, governmental, agency, non-regulatory, society, standard measure sets, and documents pertaining to individual measures. These sources are detailed in Table 1. Overall, proxies were discussed in 31 of the sources, while 8 (20.5%) did not mention proxies at all.

In the documents that provided a definition or purpose for proxies, many cited the regulatory and scientific agencies, EMA and FDA, which defined a proxy as “a person who reports an outcome as if she/he was the patient him/herself” [2] (pg 11) and a proxy-reported outcome as “a report by someone who is not the patient responding as if that person were the patient” [3] (pg 21). These agencies discourage the use of proxies, especially for symptoms and domains likely to be unknown to individuals who were not the patient, i.e., highly internal, subjective, and difficult to observe. In the discussion of proxy and observer reports for children and adolescents in the 2009 FDA guidance, pain-related behavior is cited as observable, in contrast to pain intensity, suggesting that the latter would be a domain for which the FDA would discourage proxy reporting [3].

Other sources typically defined proxies as caregivers who answered when patients were unable to answer on their own, or patients requiring assistance, such as those with cognitive impairment (Table 1). Relationship of the proxy to the patient differed among the sources, from family, caregiver, or clinician. While most of the regulatory, non-regulatory, and society documents provided broad characteristics to define the intended proxy, the individual measures occasionally provided specific definitions, such as the QOL-AD which defined proxies as those “actively involved… who lived with the patient or spent every day with them” [42].

Of the 39 sources, 12 (30.8%) provided a guidance on the perspective to be taken by the proxy, with most specifying the proxy should respond as if they were the patient. Individual measures, especially those for use with patients with dementia, often used a proxy-patient perspective (including DEMQOL [39] and QOL-AD [43]), however the QOLAS used a proxy-proxy perspective [37]. The EQ-5D has two proxy versions: one with the caregiver’s perception (proxy-proxy perspective) and one with the proxy-patient perspective [51].

Domains and symptoms that could be answered by a proxy were rarely specifically addressed by agencies or societies, with the FDA and EMA discouraging proxy reports for concepts only known by the patient, including symptoms as an example [12]. The Montreal Accord listed frequency and duration of symptoms, physical appearance, mobility, movements, limitations and restrictions to functioning, and observed behaviors as areas that a proxy with “shared experience” to the patient could report on [25]. Individual measures, particularly in dementia, were developed with attention to items that could be observed and reported by others [42, 44]. However, these also often included more subjective concepts and domains. For example, the ADRQL for Alzheimer’s disease covers social interaction, feelings and mood, and enjoyment of activities [45].

Lastly, observers were differentiated from proxies as parents, caregivers, or clinicians who can report on observed symptoms or domains [3, 22, 23]. Observer-reported outcomes (ObsROs) were defined as reports on observed behaviors or symptoms that do not include any judgments [2]. Notably, the FDA’s 2009 PRO guidance did indicate the possibility of judgment in its initial differentiation of proxy and observer outcomes: “A proxy report also is different from an observer report where the observer (e.g., clinician or caregiver), in addition to reporting his or her observation, may interpret or give an opinion based on the observation” [3] (p32). However, the Appendix to the FDA’s 2018 Patient-Focused Drug Development (PFDD) discussion document defined ObsROs as “limited to the assessment of observable signs and symptoms that can be reported from the perspective of a parent or caregiver” [14] (p17). ISPOR likewise differentiated observer raters reporting ObsRO from clinical professionals completing clinician-reported outcomes (ClinROs) [23].

Based on reviewed documents, we generated a list of considerations for the use of proxies and proxy reporting organized as follows: study design including protocol development, data collection methods, analytic methods, outcomes interpretation and reporting recommendations (Table 2). A summary follows each consideration.

**Table 2:** Checklist of consensus-based considerations for use of proxies and proxy-reported measures and reporting recommendations

Stage	Considerations	Resource
Study design	Plan and justify the use of a proxy respondent, including specifying when a proxy is needed and allowed	SPIRIT-PRO [26, 27]; NQF [19, 18]
	Provide evidence of the psychometric properties of the proxy assessment tool	SPIRIT-PRO [26, 27]; MORECare [29]
	Specify the criteria for choosing who can act as a proxy (e.g., based on contact/closeness with the patient)	Montreal Accord [25]; ISOQOL user guide [11]
	For longitudinal studies, plan for the same proxy to respond across all time points	Neuro-QoL [48]
	Specify what domains a proxy can report on, and whether judgements can be made	PCORI minimum standards [16]; Montreal Accord [25]; MORECare [29]; FDA [3]
Data Collection Methods	Describe how the proxy should respond (proxy- or patient-perspective)	ISOQOL user guide [11]
	Clear instructions for the proxy should be listed prior to the question	Neuro-QoL [48]; DEMQOL [39]
Analytic methods	Describe and justify whether and when patient-reported data might be replaced by proxy-reported data	EMA [2]
	Consider risk adjustment for proxy completion	NQF [19, 18]
	Consider sensitivity analyses to assess the potential impact of proxy-reported data on interpretation of estimates	SPIRIT-PRO [27]
Outcomes Interpretation	Consider whether the same proxy responded across all domains and time points	Neuro-QoL [48]
	Consider how proxy responses affect score interpretation and study results	CMS [15]; CONSORT PRO [28]; DEMQOL [40]
Reporting recommendations	Report on considerations above, including: Summarize who completed the proxy reports, and what proxies reported on Describe specific instructions and perspective(s) used Detail any analytic methods for interpreting results from proxies	MORECare [29]; AHRQ [52]; ISOQOL user guide [11]
	Differentiate patients and proxies in the results	MORECare [29]; CONSORT PRO [28]
	Describe how proxy responses may have affected results	CMS [15]; CONSORT PRO [28]; DEMQOL [40]

### Considerations during the study design phase

1. *Plan and justify the use of a proxy respondent, including specifying when a proxy is needed and allowed*. While the EMA and FDA discourage the use of proxies, they also note the existence of patient populations for which self-report is not possible. Many resources cite these guidelines yet indicate a proxy could be used as a last resort, if data would be otherwise missing [26, 52], and proxies should be used only when patients cannot answer [29]. At the same time, these documents acknowledge that proxies are likely to be needed [29, 52]. In clinical care or when the study context is likely to involve proxy use, the planned use of a proxy respondent should be described and justified in the study protocol [19, 26, 27].
  Although decision-making will be context- and study-specific, proxy respondents could be considered if it is anticipated that an increasing number of patients may be unable to complete questionnaires over the course of the study due to deteriorating condition [33]. Some questionnaire documentation specifies when proxies are needed: “people with MMSE ≤ 10 were unable to respond” [42]; “people with severe dementia should not respond due to large amounts of missing data” [39]. If proxies are considered due to patient cognitive impairment, the nature of the cognitive impairment should also be considered if possible. Dementia in and of itself does not necessarily mean that a patient cannot provide reliable self-report [53]. In the case of Parkinson’s disease, for example, only about 30-40% of patients progress to develop dementia, and it is often a different presentation of dementia than what results from Alzheimer’s disease (e.g., more dysexecutive and less amnestic) [54]. In any case, planning and justification for proxy use at the study design phase, rather than during the study itself, should be undertaken, and clearly indicated in the study design. Clear specification of when to use proxy rather than patient respondents can promote standardization and more consistent decision-making.
2. *Provide evidence of the psychometric properties of the proxy assessment tool.* If using proxy-reports, it is recommended the reliability and validity of the assessment measured be described, or evidence should be cited on the validity [26, 27, 29]. In general, and consistent with guidelines such as SPIRIT-PRO [26], information on the psychometric properties of the assessment tool should be provided, as they would be for all PRO measures. The Agency for Healthcare Research and Quality (AHRQ) extends this to recommend the extent of patient-proxy agreement on the measure be established in advance of using proxy-reports [52]. In some instances, agreement on the measure may be unknown or impossible to evaluate, such as if patients never self-report. At a minimum, the study protocol should address the expected patient-proxy agreement and researchers should consider the potential effect on study results.
3. *Specify the criteria for choosing who can act as a proxy (e.g., based on contact/closeness with the patient).* Who can act as a proxy differs by document source, from someone having a shared experience [55], to an actively involved caregiver who lives with or spends every day with the patient [42], to nursing staff or family who are well acquainted with the patient [38, 44]. The International Consortium for Health Outcomes Measurement (ICHOM) standard outcome set for stroke indicates proxies, clinicians, or abstraction from the medical records would all be appropriate for completing missing outcomes data [31]. Because the proxy is reporting about the patient, the proxy should be identified in terms of their relationship to the patient [11]. It is important to clarify who is reporting and use consistent terminology (i.e. proxy reporting, observer reporting, or clinician reporting). Beyond this and in general, when specifying and justifying the criteria for who can serve as a proxy, it is important to consider factors which may influence proxy raters, such as their degree of emotional involvement or neutrality, and how that may affect study results [29]. Additionally, practical considerations may play a role, such as the accessibility of the person at the time of outcome assessment, whether a consistent proxy must be used over time or if a variable proxy can be used in certain contexts (e.g. clinical contexts), and these should be discussed, if applicable.
4. *For longitudinal studies, plan for the same proxy to respond across all time points.* Neuro-QoL recommends that the same proxy should respond across multiple assessments as different proxies may have different viewpoints and frames of reference [48]. Attempting to have the same proxy respond for the duration of the longitudinal study may limit variability when comparing results across time.
5. *Specify what domains a proxy can report on, and whether judgements can be made.* Document sources indicated that proxies should not make judgements and proxy reporting should be limited to observable events or behaviors to avoid bias, as it may be difficult for a proxy to know how the patient is feeling [16]. FDA discourages all use since “proxy reporting can lead to inappropriate inferences and may not be reflective of what a patient may be truly thinking or feeling” [12] (pg. 17), with the suggestion that ObsROs rather than ProxROs be used when it is impossible to collect “valid and reliable self-report data from the patient” [14] (pg. 17). Observers are often taught what to observe to form the rating for the ObsRO [24]. The Montreal Accord stipulates that proxies, with a shared or observed experience, are a type of observer and can report on frequency and duration of symptoms, physical impairments and function, and behavior but should not report on perceptions of health and QoL [25]. Generally, an observer would not make a judgement when providing an ObsRO [13]; thus, explicit discussion of whether judgement is involved can be useful in differentiating proxy- and observer-reported outcomes.

While there is no clear consensus on what a proxy can report on, and whether judgements can be made, researchers should consider the possible degree of proxy bias across different domains in the context of the information they are trying to capture. As ratings are influenced by the perspective and individual, this information is useful in interpreting the validity of results. It is important to outline what a proxy can report on and whether judgements can be made.

### Considerations during data collection

1. *Describe how the proxy should respond (proxy- or patient-perspective).* Most commonly, the proxy-patient perspective was indicated, where the proxy responds as if they were the patient (Table 1). However, the proxy-proxy perspective could also be appropriate, where the proxy provides their own perspective on the patient [37]. The chosen perspective should be considered in the context of the study as well as the requirements of the chosen measure, and the choice of perspective should be documented. It should also be clear whether judgements should be provided for all questions, or observable domains only.
2. *Clear instructions for the proxy should be listed prior to the question.* Providing instructions to the proxy can help them complete the measure. In addition, as noted above, proxy perspectives are not always recorded; including instructions can assist proxies with answering questions from the perspective of interest, leading to better alignment of how measures are described and how they are completed in practice. Instructions should be pre-tested for readability and comprehensibility to maximize their usefulness. As an example, the DEMQOL measure provides a detailed set of instructions to the interviewer, including suggestions for how to handle respondent queries [39].

### Considerations for the analytic methods

1. *Describe and justify whether and when patient-reported data might be replaced by proxy-reported data.* The clinical study protocol should define clear rules as to whether and when patient-reported data may be replaced with proxy-reported data, along with justification [2]. For instance, at the time that a patient with a progressive neurological condition develops dementia. This may not always be appropriate, and where guidance from specific measures is available it should be followed. For example, DEMQOL is clear that DEMQOL-Proxy responses should not be substituted for missing DEMQOL responses [40, 56]. Researchers using the DEMQOL should therefore follow this guidance. In addition, a paper provides a crosswalk between the two [40].
2. *Consider risk adjustment for proxy completion.* Risk adjustment may need to incorporate differences in PRO-values related to proxy responses [18, 19]. Adjusting for proxy-response in statistical modeling of outcomes may be appropriate.

*Consider sensitivity analyses to assess the potential impact of proxy-reported data on estimates.* If justification has been provided for replacing patient-reported data with proxy-reported data, consider sensitivity analyses such as stratifying data analysis by patient- versus proxy-reported data, or excluding proxy-reported data from the analysis to evaluate if results remain consistent [27].

### Considerations for outcomes interpretation

1. *Consider whether the same proxy responded across all domains and time points.* Administration guidelines for Neuro-QoL specify the same proxy should respond across multiple assessments as different proxies may have different viewpoints [48]. Similarly, FDA documentation says “every effort should be made to ensure that all observer-reported assessments for a given subject are completed by the same individual throughout the study” [14] (pg. 15). If this is not the case in the study, results should be interpreted in light of this possible bias.
2. *Consider how proxy responses affect score interpretation and study results.* The utilization of proxy-reports and the potential bias or effect on the results should be addressed [15, 28]. Discuss the results of any sensitivity analyses, and if results are inconsistent, discuss how proxies may have affected interpretation of study results. The ISOQOL user guide suggests discussing as a limitation that the proxy may have a difficult time distinguishing between how the patient would respond versus their own perception of the patient’s status [11].

### Reporting recommendations

All of the above considerations should be described, however the following points summarize recommendations for minimum information that should be reported in studies that include proxy reports.

1. *Summarize who completed the proxy reports, and what proxies reported on*. In the methods, describe the process of obtaining proxy reports, including specifying when a proxy was allowed, and information on proxy selection criteria. Describe who completed the proxy-reported measures, e.g., a professional caregiver or a close family member (who should be defined in terms of their relationship to the patient, e.g., spouse/partner or child) [52]. In addition, if information such as relevant proxy-related factors that are seen as possibly influencing proxy raters are collected, then this should be reported as well. If different proxies responded across the life of the study for a patient, this should be described [48]. Discuss if proxies could report on any domain, or if any judgements were made by the proxy.
2. *Describe specific instructions and perspective(s) used.* To aid in interpreting results, information on what perspective the proxy was asked to use and any specific instructions to the proxy for measure completion should be summarized.
3. *Detail any analytic methods for interpreting results from proxies*. Consider and address whether the data can be pooled within and between patients or proxy respondents. Any analytic methods, such as adjustment for proxy report, or sensitivity analyses, should be described.
4. *Differentiate patients and proxies in the results*. Provide a summary of patient and proxy reports and highlight any differences in results when reported by patients and reported by proxies [28, 29].
5. *Describe how proxy responses may have affected results*. A thoughtful discussion on whether and how proxy responses may have affected the interpretation of results should be included [15, 28, 40].

## Discussion

Our study summarizes the findings of a targeted review by ISOQOL’s Proxy Task Force to identify definitions and practices regarding the use of proxies and proxy-reported measures for adults. After extracting and summarizing 39 sources of proxy-related documents, a summary checklist of considerations was developed for the use of proxies and proxy-reported data in the study design phase, during data collection and analysis, and when interpreting and reporting results.

Overall, our targeted review identified a number of areas of convergence and divergence, as well as areas in which advice is absent or limited, in the available guidance from agencies and professional societies related to proxies. FDA discourages the use of proxy-reports [3, 12, 14], and many other agencies cite this discouragement but suggest occasions when proxy-reports may be necessary to avoid loss of data [2, 26, 52]. Generally, there was consensus among sources that proxies may be necessary when patients are unable to self-report. Who can act as a proxy differed considerably between sources from “someone other than the patient” [3] and non-clinical caregivers [16, 24], to close family [52], and clinicians and physicians [28, 29, 33]. Whether a proxy should respond on behalf of the patient (proxy-patient perspective) or on behalf of themselves (proxy-proxy perspective) was rarely specified, despite perspective taken in providing the report being part of the definition of a proxy in some cases [2, 3]. Areas that a proxy could report on also differed among sources, with some documents suggesting that proxy reports focus on observed symptoms or domains, or otherwise differentiating between proxy-reports versus observer-reports. Surprisingly, many measure sets or measures in the condition-specific areas where we anticipated high rates of proxy-reports, such as cancer, stroke, and palliative care, did not address proxy responses at all [30, 31, 47, 50]. Issues of missing data and proxy response are both recognized as threats to the validity of PROs [19], and the development of guidelines for addressing these issues and setting standards for proxy-reported measures is a high-priority need for the field of PROs in research and clinical practice. While no clear consensus was achieved following our targeted review, our study utilized the reviewed documents to develop a checklist of considerations (see Table 2) to strengthen the inclusion, rigor, and interpretability of data provided by proxies, including data based on proxy-reported measures.

As the Task Force’s initial large-scale review uncovered, there are many issues relating to proxy reporting on which further clarity is required, including who can report as a proxy and how to differentiate proxy reports from other COAs [7]. As indicated above, our current analysis similarly noted variability in these issues and our work highlights the necessity for clarity surrounding multiple issues, including but not limited to when to use a proxy, who can be a proxy, what a proxy can report on, and how the proxy should respond.

First, when considering PRO collection in research or clinical care, it must be determined if proxy use is justified. Within the clinical context, whether proxies should be used and when they could be needed must be carefully considered. Only a few instruments such as the QOL-AD and DEMQOL provided clear guidelines for when proxies should be used [39, 42]. Clear justification, and the reporting of this justification, is important for robust study design and to facilitate more interpretable results. Our targeted review identified that most guidance relates to proxies reporting only on objective domains, without making judgments. However, following only this guidance would have profound implications for the evaluation of health-related QoL in patients who are unable to self-report, and implies the invalidation of several well-developed measures such as the DEMQOL, which have addressed these issues methodologically [39, 40]. There is thus a need for additional guidance to support the capture of subjective patient health in populations unable to self-report [29].

Second, who can be a proxy must be considered. This may vary across clinical contexts. In the rehabilitation literature, a proxy must have a shared experience with the patient [25]. In other clinical conditions, such as dementia where a patient is living in a nursing home or aged care setting, a clinician or staff member may have more contact with the patient than a family member, and may be the most appropriate proxy [38, 44]. It is important to consider both the study context and what is expected from the proxy when determining who can act as a proxy, including whether repeated measurement is required, and to collect information so it can be described and reported appropriately.

Third, what proxies can or should report on should be stated. There was a lack of clarity and precision surrounding the terminology used for proxies that seemed to differ by research versus clinical contexts. The FDA and EMA differentiated proxies, reporting as if they are the patient, from observers (e.g., clinician or caregiver) as reporting on events/behaviors that are observed [2, 3, 12, 14]. Using only these regulatory definitions would present substantial challenges for categorizing proxy reporting that uses the proxy-proxy perspective. Non-regulatory standards used terms interchangeably: the Patient-Centered Outcomes Research Institute (PCORI) minimum standards used proxy and caregiver interchangeably [16], and Organisation for Economic Cooperation and Development (OECD) used proxy and observer interchangeably [17]. Some clinical sources differentiated observers and ObsRO as those based on observed behaviors [23], and proxies and ProxROs as based on shared experience [25]. In the case of the latter, a proxy is conceptualized as a special kind of observer, with a shared experience. Overall, there is a need for these terms to be clearly defined and differentiated.

This discrepancy further raises the question of proxy use in certain clinical contexts, such as dementia, where self-report may not be possible [57]. Patients and caregivers or family members have different points of reference, further complicating the reliability of self- versus proxy-reports. There are recommendations to collect proxy and patient data simultaneously (e.g., SPIRIT-PRO) [27], however there is a lack of consensus on how to handle simultaneously collected proxy and patient data. DEMQOL provides a crosswalk of proxy- to patient-reports, but few measures have done this work [40]. For patients with cognitive deficits or dementia, there has been much consideration over whether there is a threshold of cognitive skills needed to self-report. Questions still remain such as whether evidence should be required that people cannot self-report, before assuming they cannot. Another question is whether this should differ by domain as proxies may be less able to report on unobservable behaviors or feelings, even if the patient has more severe cognitive deficits [58]. Researchers should consider and balance prioritization of the patient’s self-report with the measurement error introduced by having data from some patients or time points provided by different raters. For example, in a longitudinal study of participants at risk of developing dementia, researchers would need to decide whether to have proxy-report data from everyone (potentially reducing measurement error but limiting the constructs that can be assessed) versus obtaining self-report data from those who can provide it (but introducing measurement error by having proxy raters for those who cannot provide self-report).

Fourth, it should be elucidated how the proxy will be instructed to respond. The perspective should be stated, whether the proxy is reporting on behalf of the patient, or on their own. This is important on a theoretical basis to enable understanding and interpretation of the results. This can be achieved through clear instructions to proxies when completing the measure. With increasing cognitive impairment, and especially with concomitant neuropsychiatric challenges such as delusional thinking, the proxy-patient perspective (responding from the patient’s perspective) becomes increasingly difficult, and the proxy-proxy perspective may be more useful and reliable.

Future research and guidance should focus on clarifying these identified issues including providing a consistent definition of proxy reports, and guidance on when to use a proxy, who can be a proxy, what a proxy can report on, and how the proxy should respond. However, given the wide range of health conditions and studies that could incorporate proxy responses, a one-size-fits-all approach to including proxies in studies or clinical care is inappropriate. Whether proxies will be included, who a proxy can be, what they can report on, and in which perspective will differ by study and clinical context. How the data will be analyzed and interpreted will also likely differ by study and clinical context. MORECare identified determining the reliability of proxy-reported measures as an area of future research, specifying that knowing what influences proxy reporting will ultimately improve the reliability and validity of proxy measures [29]. However, the patient-proxy agreement literature has investigated predictors of improved patient-proxy agreement to little avail [6, 59, 60]. More research is necessary in the area of statistical methods for the analysis of proxy-reported data. Some studies have found adjusting for proxy-reports in the analysis is not enough to account for proxy-introduced bias [61] while others have demonstrated the benefit in accounting for proxies in PRO group-level analyses [62, 63]. Future research is needed in the analysis and risk adjustment of proxy-reported data. The checklist in this paper can guide researchers until further research is able to support a more nuanced approach.

Our study summarized the guidance on proxy reporting, and provided a preliminary list of considerations for the use of proxies and proxy-reported measures. There are some limitations that warrant discussion. First, this was a targeted review, and not intended to be a comprehensive evaluation. Our checklist of considerations was not developed as a comprehensive list of recommendations, but an initial summary of emerging best practices for collecting and reporting data from proxies. There are other conditions where expected use of proxy reports is high, such as traumatic brain injury, which were not included in our study. The conditions chosen were selected to provide examples, and do not represent all conditions where proxy use may be high. Second, our study focuses on adults. Much has been written about child-parent proxy reports, with some guidance already available [64, 65]. There are clear limitations to the generalizability of guidelines and research on proxy-reports for children to proxy-reports for adults. For example, the ISPOR PRO good research practices report discusses age and developmental criteria regarding child ability to self-report [64]. Our study’s focus on adult health research is therefore appropriate and addresses an unmet need in the literature. Third, proxy-reported bias is complex and differs by domain, condition severity, characteristics of the proxy and patient, and change over time [66–69] which further complicates the ability to create a one-size-fits-all approach to standardizing guidelines for proxy-reporting. Fourth, our targeted review largely focuses on proxy-reports in research studies. However, the issue of proxy reports is also recognized in clinical care. For example, the PCORI-funded Users’ Guide to Integrating Patient-Reported Outcomes in Electronic Health Records notes that proxies can be used when patients are unable to complete PROs and discusses options for recording information about data collection in the PRO-EHR system [70]. Similarly, Murtagh et al.’s [8] validation of the Integrated Palliative care Outcome Scale (IPOS) cited “maximum flexibility for clinical use” when evaluating both proxy and patient versions. At a minimum, if family members or caregivers accompany patients to office visits and complete all paperwork, including PROs, on their behalf, this should be captured as it can subsequently affect research involving clinical data or interpretation of PROs at the individual-level. As much as possible, efforts should be made to realize this occurs and follow our suggested considerations for providing clear instructions to proxies, capturing who responds to outcome measures, and establishing appropriate study methods for accounting for proxy-reports. Whether proxies are completing PROs or a proxy-reported measure, how the individual is provided with the instructions and the perspective to take should be clear. Fifth, considerations listed in our paper are based on group-level analyses which can accept more uncertainty and measurement error than interpretation at the individual-level. Lastly, proxies were not included in the Task Force or the development of these considerations. Proxies should be considered for participation in future consensus development initiatives.

## Conclusions

In conclusion, this targeted review highlighted the lack of clarity and precision in the area of proxy-reporting for adult populations. This paper summarizes available guidance, provides preliminary considerations for including and reporting on use of proxies, and highlights areas where further guidance is necessary. The development of a consensus-based checklist of considerations can support stakeholders who collect and use patient-centered data for research and clinical care. Addressing these issues will lead to better standards in the field and ultimately higher-quality research and clearer interpretation of research findings to inform clinical utilization.

## Supplementary Information

**Additional file 1.** Data Extraction Form.

## Abbreviations

AHRQ: The Agency for Healthcare Research and Quality
ClinRO: Clinician-reported outcome
COA: Clinical outcome assessment
EMA: European Medicines Agency
FDA: US Food and Drug Administration
ICHOM: International Consortium for Health Outcomes Measurement
IPOS: Integrated palliative care outcome scale
ISOQOL: International Society for Quality of Life
ObsRO: Observer-reported outcome
OECD: Organisation for Economic Cooperation and Development
PCORI: Patient-Centered Outcomes Research Institute
PRO: Patient-reported outcome
ProxRO: Proxy-reported outcome
QoL: Quality of life

## References

[1] About FDA. Clinical outcome assessment (COA): frequently asked questions. https://www.fda.gov/about-fda/clinical-outcome-assessment-coa-frequently-asked-questions. Accessed 31 Mar 2023

[2] European Medicines Agency (2016) Appendix 2 to the guideline on the evaluation of anticancer medicinal products in man. The use of patient-reported outcome (PRO) measures in oncology studies. https://www.ema.europa.eu/en/documents/other/appendix-2-guideline-evaluation-anticancer-medicinal-products-man_en.pdf. Accessed 31 Mar 2023

[3] U.S. Department of Health and Human Services, Food and Drug Administration (2009) Guidance for industry. Patient-reported outcome measures: use in medical product development to support labeling claims. https://www.fda.gov/media/77832/download. Accessed 31 Mar 2023

[4] Gliklich RE, DNA, Leavy MB (eds) (2014) Registries for evaluating patient outcomes: a user’s guide [internet]. 3rd edition. Rockville, MD: Agency for Healthcare Research and Quality (US); 2014 Apr. Report No.: 13(14)-EHC111 24945055

[5] Rand SCJ (2015) Quality and outcomes of person-centred care policy research unit. Using proxies to assess quality of life: a review of the issues and challenges. https://www.pssru.ac.uk/pub/4980.pdf. Accessed 31 Mar 2023

[6] Sneeuw KC, Sprangers MA, Aaronson NK (2002). The role of health care providers and significant others in evaluating the quality of life of patients with chronic disease. J Clin Epidemiol.

[7] Roydhouse JK, Cohen ML, Eshoj HR, Corsini N, Yucel E, Rutherford C (2022). The use of proxies and proxy-reported measures: a report of the international society for quality of life research (ISOQOL) proxy task force. Qual Life Res.

[8] Murtagh FE, Ramsenthaler C, Firth A, Groeneveld EI, Lovell N, Simon ST (2019). A brief, patient- and proxy-reported outcome measure in advanced illness: validity, reliability and responsiveness of the integrated palliative care outcome scale (IPOS). Palliat Med.

[9] FDA-NIH Biomarker Working Group (2016) BEST (Biomarkers, EndpointS, and other Tools) Resource [Internet]. Glossary, clinical outcome assessment (2016 Jan 28 [Updated 2021 Nov 29]). https://www.ncbi.nlm.nih.gov/books/NBK338448/def-item/glossary.clinical-outcome-assessment/. Accessed 31 Mar 2023

[10] Pickard AS, Knight SJ (2005). Proxy evaluation of health-related quality of life: a conceptual framework for understanding multiple proxy perspectives. Med Care.

[11] International Society for Quality of Life Research (prepared by Aaronson N, Elliott T, Greenhalgh J, Halyard M, Hess R, Miller D, Reeve B, Santana M, Snyder C). User’s Guide to Implementing Patient-Reported Outcomes Assessment in Clinical Practice, Version: January 2015. https://www.isoqol.org/wp-content/uploads/2019/09/2015UsersGuide-Version2.pdf. Accessed 31 Mar 2023

[12] U.S. Department of Health and Human Services, Food and Drug Administration (2022) Patient-focused drug development: methods to identify what is important to patients. https://www.fda.gov/media/131230/download. Accessed 31 Mar 2023

[13] U.S. Department of Health and Human Services. Food and Drug Administration (2022) Patient-focused drug development: selecting, developing, or modifying fit-for-purpose clinical outcome assessments. Guidance for industry, food and drug administration staff, and other stakeholders. Draft Guidance. https://www.fda.gov/regulatory-information/search-fda-guidance-documents/patient-focused-drug-development-selecting-developing-or-modifying-fit-purpose-clinical-outcome. Accessed 31 Mar 2023

[14] U.S. Food and Drug Administration. (2018) Patient-Focused drug development guidance public workshop. Methods to identify what is important to patients and select, develop, or modify fit-for-purpose clinical outcomes assessments. Workshop date: October 15–16, 2018. https://www.fda.gov/media/116281/download. Accessed 31 Mar 2023

[15] Centers for Medicare and Medicaid Services (2022) Measures management system (MMS) hub. Supplemental material: patient-reported outcome measures. https://mmshub.cms.gov/sites/default/files/Patient-Reported-Outcome-Measures.pdf. Accessed 31 Mar 2023

[16] Acaster SCT, Lloyd A (2012). Patient Centered Outcomes Research Institute (PCORI). Development of a methodological standard report: Topic #3. The design and selection of patient-reported outcomes measures (PROMs) for use in patient centered outcomes research. https://www.pcori.org/assets/The-Design-and-Selection-of-Patient-Reported-Outcomes-Measures-for-Use-in-Patient-Centered-Outcomes-Research1.pdf. Accessed 31 Mar 2023

[17] Organisation for Economic Co-operation and Development (OECD) (2017) Recommendations to OECD ministers of health from the high level reflection group on the future of health statistics. Strengthening the international comparison of health system performance through patient-reported indicators. https://www.oecd.org/health/Recommendations-from-high-level-reflection-group-on-the-future-of-health-statistics.pdf. Accessed 31 Mar 2023

[18] National Quality Forum (2012) Patient reported outcomes (PROs) in performance measurement. https://www.qualityforum.org/Publications/2012/12/Patient-Reported_Outcomes_in_Performance_Measurement.aspx. Accessed 31 Mar 2023

[19] National Quality Forum (2020) Building a roadmap from patient-reported outcome measures to patient-reported outcome performance measures. https://www.qualityforum.org/ProjectDescription.aspx?projectID=93898. Accessed 31 Mar 2023

[20] Hopper L, Bowen C (2021) Development of a model for the national dementia registry. Dublin: Dublin City University. https://www.dcu.ie/sites/default/files/inline-files/development-of-a-model-for-the-national-dementia-registry.pdf. Accessed 31 Mar 2023

[21] Reeve BB, Wyrwich KW, Wu AW, Velikova G, Terwee CB, Snyder CF (2013). ISOQOL recommends minimum standards for patient-reported outcome measures used in patient-centered outcomes and comparative effectiveness research. Qual Life Res.

[22] Mayo NE (2015). Dictionary of quality of life and health outcomes measurement.

[23] Walton MK, Powers JH, Hobart J, Patrick D, Marquis P, Vamvakas S (2015). Clinical outcome assessments: conceptual foundation-report of the ISPOR clinical outcomes assessment—emerging good practices for outcomes research task force. Value Health.

[24] Benjamin K, Vernon MK, Patrick DL, Perfetto E, Nestler-Parr S, Burke L (2017). Patient-reported outcome and observer-reported outcome assessment in rare disease clinical trials: an ISPOR COA emerging good practices task force report. Value Health.

[25] Mayo NE, Figueiredo S, Ahmed S, Bartlett SJ (2017). Montreal accord on patient-reported outcomes (PROs) use series—paper 2: terminology proposed to measure what matters in health. J Clin Epidemiol.

[26] Calvert M, Kyte D, Mercieca-Bebber R, Slade A, Chan AW, King MT (2018). Guidelines for inclusion of patient-reported outcomes in clinical trial protocols: the SPIRIT-PRO extension. JAMA.

[27] Calvert M, King M, Mercieca-Bebber R, Aiyegbusi O, Kyte D, Slade A (2021). SPIRIT-PRO Extension explanation and elaboration: guidelines for inclusion of patient-reported outcomes in protocols of clinical trials. BMJ Open.

[28] Calvert M, Blazeby J, Altman DG, Revicki DA, Moher D, Brundage MD (2013). Reporting of patient-reported outcomes in randomized trials: the CONSORT PRO extension. JAMA.

[29] Evans CJ, Benalia H, Preston NJ, Grande G, Gysels M, Short V (2013). The selection and use of outcome measures in palliative and end-of-life care research: the MORECare International Consensus Workshop. J Pain Symptom Manag.

[30] The International Consortium for Health Outcomes Measurement (ICHOM). Oncology. https://www.ichom.org/patient-centered-outcome-measures/. Accessed 31 Mar 2023

[31] The International Consortium for Health Outcomes Measurement (ICHOM). Stroke. Data Collection Reference Guide (2018). https://ichom.org/files/medical-conditions/stroke/stroke-reference-guide.pdf. Accessed 31 Mar 2023

[32] Core Outcome Measures in Effectiveness Trials (COMET). https://www.comet-initiative.org/. Accessed 31 Mar 2023

[33] Wintner LM, Sztankay M, Giesinger JM, Aaronson N, Bottomley A, Velikova G, Verdonck de Leeuw I, van de Poll-Franse L, Groenvold M, Petersen MA, Holzner B (2016) European organization for research and treatment of cancer (EORTC) quality of life group. Manual for the use of EORTC measures in daily clinical practice. https://www.eortc.org/app/uploads/sites/2/2018/02/EORTC_QLQ_Clinical_Practice_User_Manual-1.0.pdf. Accessed 31 Mar 2023 10.1016/j.ejca.2016.08.024 27721057

[34] EORTC Quality of Life Group (2002) Guidelines for assessing quality of life in EORTC clinical trials. https://www.eortc.org/app/uploads/sites/2/2018/02/clinical_trials__guidelines_qol.pdf. Accessed 31 Mar 2023.

[35] FACIT group. https://www.facit.org/. Accessed 31 Mar 2023

[36] PROMIS (Patient-Reported Outcomes Measurement Information System). https://www.healthmeasures.net/explore-measurement-systems/promis. Accessed 31 Mar 2023

[37] Selai CE, Trimble MR, Rossor MN, Harvey RJ (2001). Assessing quality of life in dementia: preliminary psychometric testing of the quality of life assessment schedule (QOLAS). Neuropsychol Rehabil.

[38] Terada S, Ishizu H, Fujisawa Y, Fujita D, Yokota O, Nakashima H (2002). Development and evaluation of a health-related quality of life questionnaire for the elderly with dementia in Japan. Int J Geriatr Psychiatry.

[39] Smith SC, Lamping DL, Banerjee S, Harwood R, Foley B, Smith P (2005). Measurement of health-related quality of life for people with dementia: development of a new instrument (DEMQOL) and an evaluation of current methodology. Health Technol Assess.

[40] Smith SC, Hendriks AAJ, Cano SJ, Black N (2020). Proxy reporting of health-related quality of life for people with dementia: a psychometric solution. Health Qual Life Outcomes.

[41] Ready RE, Ott BR, Grace J, Fernandez I (2002). The Cornell-Brown scale for quality of life in dementia. Alzheimer Dis Assoc Disord.

[42] Logsdon RG, Gibbons LE, McCurry SM, Teri L (2002). Assessing quality of life in older adults with cognitive impairment. Psychosom Med.

[43] Logsdon RG, Gibbons LG, McCurry SM, Teri L (1999). Quality of life in Alzheimer's disease: patient and caregiver reports. J Ment Health Aging.

[44] Kasper JD, Black BS, Shore AD, Rabins PV (2009). Evaluation of the validity and reliability of the Alzheimer disease-related quality of life assessment instrument. Alzheimer Dis Assoc Disord.

[45] Rabins PV, Kasper JD, Kleinman L, Black BS, Patrick DL (1999). Concepts and methods in the development of the ADRQL: an instrument for assessing health-related quality of life in persons with Alzheimer’s disease. J Ment Health Aging.

[46] Cleeland CS (2016) The M.D. Anderson symptom inventory. User Guide. Version 1. https://www.mdanderson.org/content/dam/mdanderson/documents/Departments-and-Divisions/Symptom-Research/MDASI_userguide.pdf. Accessed 31 Mar 2023

[47] EORTC quality of life, questionnaires. https://qol.eortc.org/questionnaires/. Accessed 31 Mar 2023

[48] Neuro-QoL. Obtain and Administer Measures. https://www.healthmeasures.net/explore-measurement-systems/neuro-qol/obtain-and-administer-measures. Accessed 23 Mar 2023

[49] Adult Sickle Cell Quality of Life Measurement Information System (ASCQ-Me). Obtain and Administer Measures. https://www.healthmeasures.net/explore-measurement-systems/ascq-me/obtain-and-administer-measures. Accessed 31 Mar 2023

[50] Palliative Care Research Cooperative Group. Palliative Care Measurement Tool Library. https://palliativecareresearch.org/corescenters/measurement-core/Palliative-Care-Measurement-Tool-Library. Accessed 31 Mar 2023

[51] EQ-5D. https://euroqol.org/eq-5d-instruments/ Accessed 31 Mar 2023

[52] Agency for Healthcare Research and Quality. https://www.ahrq.gov/cahps/surveys-guidance/cg/index.html. Accessed 31 Mar 2023 10.1080/15360280802537332 21923316

[53] Cohen ML, Hula WD (2020). Patient-reported outcomes and evidence-based practice in speech-language pathology. Am J Speech Lang Pathol.

[54] Janvin CC, Larsen JP, Salmon DP, Galasko D, Hugdahl K, Aarsland D (2006). Cognitive profiles of individual patients with Parkinson's disease and dementia: comparison with dementia with Lewy bodies and Alzheimer's disease. Mov Disord.

[55] Bingham CO, Noonan VK, Auger C, Feldman DE, Ahmed S, Bartlett SJ (2017). Montreal Accord on Patient-Reported Outcomes (PROs) use series—paper 4: patient-reported outcomes can inform clinical decision making in chronic care. J Clin Epidemiol.

[56] DEMQOL and DEMQOL-Proxy—Interviewer Manual. Instructions for administration. https://www.bsms.ac.uk/_pdf/cds/interviewer-manual.pdf. Accessed 31 Mar 2023

[57] Smith SC, Hendriks AJ, Regan J, Black N (2018). A novel method of proxy reporting questionnaire based measures of health-related quality of life of people with dementia in residential care: a psychometric evaluation. Patient Relat Outcome Meas.

[58] Barrett AM (2010). Rose-colored answers: neuropsychological deficits and patient-reported outcomes after stroke. Behav Neurol.

[59] Lapin BR, Thompson NR, Schuster A, Katzan IL (2021). Magnitude and variability of stroke patient-proxy disagreement across multiple health domains. Arch Phys Med Rehabil.

[60] Oczkowski C, O'Donnell M (2010). Reliability of proxy respondents for patients with stroke: a systematic review. J Stroke Cerebrovasc Dis.

[61] Roydhouse JK, Gutman R, Keating NL, Mor V, Wilson IB (2018). Proxy and patient reports of health-related quality of life in a national cancer survey. Health Qual Life Outcomes.

[62] Lapin B, Thompson N, Schuster A, Katzan IL (2021). Optimal methods for reducing proxy-introduced bias on patient-reported outcome measurements for group-level analyses. Circ Cardiovasc Qual Outcomes.

[63] Shardell M, Hicks GE (2014). Statistical analysis with missing exposure data measured by proxy respondents: a misclassification problem within a missing-data problem. Stat Med.

[64] Matza LS, Patrick DL, Riley AW, Alexander JJ, Rajmil L, Pleil AM (2013). Pediatric patient-reported outcome instruments for research to support medical product labeling: report of the ISPOR PRO good research practices for the assessment of children and adolescents task force. Value Health.

[65] Mpundu-Kaambwa C, Bulamu N, Lines L, Chen G, Dalziel K, Devlin N (2022). A systematic review of international guidance for self-report and proxy completion of child-specific utility instruments. Value Health.

[66] Kroenke K, Stump TE, Monahan PO (2022). Agreement between older adult patient and caregiver proxy symptom reports. J Patient Rep Outcomes.

[67] Neumann PJ, Araki SS, Gutterman EM (2000). The use of proxy respondents in studies of older adults: lessons, challenges, and opportunities. J Am Geriatr Soc.

[68] Howland M, Allan KC, Carlton CE, Tatsuoka C, Smyth KA, Sajatovic M (2017). Patient-rated versus proxy-rated cognitive and functional measures in older adults. Patient Relat Outcome Meas.

[69] Pickard AS, Johnson JA, Feeny DH, Shuaib A, Carriere KC, Nasser AM (2004). Agreement between patient and proxy assessments of health-related quality of life after stroke using the EQ-5D and Health Utilities Index. Stroke.

[70] Snyder C, Wu AW (eds) (2017) Users’ guide to integrating patient-reported outcomes in electronic health records. Baltimore, MD: Johns Hopkins University. Funded by Patient-Centered Outcomes Research Institute (PCORI); JHU Contract No. 10.01.14 TO2 08.01.15. https://www.pcori.org/sites/default/files/PCORI-JHU-Users-Guide-To-Integrating-Patient-Reported-Outcomes-in-Electronic-Health-Records.pdf. Accessed 31 Mar 2023

