# Supplementary material for: Development of consensus-based considerations for use of adult proxy reporting: an ISOQOL task force initiative
Source: J Patient Rep Outcomes. 2023 Jun 2;7:52. doi: 10.1186/s41687-023-00588-6 (PMC10238331; doi:10.1186/s41687-023-00588-6)
Supplement: Supplementary file 1 — Additional file 1. Data Extraction Form. [file 41687_2023_588_MOESM1_ESM.docx]

**Appendix**

**Additional file 1: Data Extraction Form**

Resource- Specify:

Resource Link/Location:

**General Instructions: Please copy and paste relevant text where possible. If the resource provides relevant examples, these can be included as pasted text below the pertinent Y/N questions. If the resource mentions specific measures, please note these and then create a new form and extract information from those measures.**

**RESOURCE SECTION**

Purpose of Resource: Describe the purpose of the resource. If it is a measure, indicate if it has a goal of use (e.g., research; screening; clinical decision-making):

Screening Question: Does resource discuss proxies?

*Provide x or otherwise indicate one of the following responses*

No:

Yes:

If No, proceed to Observer section only if relevant (e.g., regulatory document, society document)

If Yes, proceed to Proxy section

**PROXY SECTION**

Preliminary Question: Does resource provide ANY of the following: a definition of a proxy; instructions for proxies or for involving a proxy; purpose of proxy involvement?

*Provide x or otherwise indicate one of the following responses*

No to ALL:

Yes to ANY:

I. Definition of Proxy: Does resource provide a definition of a proxy (e.g., who the person can be, any characteristics, etc.)?

*Provide x or otherwise indicate one of the following responses*

No:

Yes:

If No, proceed to next section

If Yes, answer questions below

1. Is relationship to patient specified? Y/N

If Yes, list relationship(s) specified:

1. Are any other characteristics specified? Y/N

If Yes, list characteristic(s) specified:

1. Does definition discuss perspective? Y/N

If Yes, list perspective(s) specified:

II. Components of Measure: What is included in the measure (e.g. instructions, types of domains, judgment involved)

1. Are there any instructions regarding proxy use (e.g. when to use, perspectives for proxy to take, etc.)? Y/N

If Yes, describe instruction(s):

1. If applicable (e.g., a specific measure is discussed): What types of domains are included in the measure? List each domain in the table below and indicate if these domains are directly observable or concrete (e.g., walking, crying) or not directly observable or concrete (e.g., feel sad, feel satisfied). For observable domains, indicate if the measure has the proxy make judgment is any aspect of these domains (e.g., ‘walks well’ rather than ‘walks 5 times’). Add rows as necessary.

| Domain | Aspects comprising domain | All aspects observable: Y/N | Judgment (for observable only): Y/N | Comments (if any) |
| --- | --- | --- | --- | --- |
|  |  |  |  |  |
|  |  |  |  |  |
|  |  |  |  |  |
|  |  |  |  |  |
|  |  |  |  |  |

1. Other (e.g., a specific measure is not discussed but other information pertaining to proxy measures appears in the resource). If applicable, paste relevant text here:

**OBSERVER SECTION – REGULATORY OR SOCIETY DOCUMENTS ONLY**

Preliminary Question: Does resource provide ANY of the following: a definition of an observer; a definition of an observer measure?

*Provide x or otherwise indicate one of the following responses*

No to ALL:

Yes to ANY:

If No, stop

If Yes, proceed

I. Definition of Observer: Does resource provide a definition of the observer (e.g., who the person can be, any characteristics, etc.?)

*Provide x or otherwise indicate one of the following responses*

No:

Yes:

If No, proceed to next section

If Yes, answer questions below

1. Is relationship to patient specified? Y/N

If Yes, list relationship(s) specified:

1. Are any other characteristics specified? Y/N

If Yes, list characteristic(s) specified:

II. Definition of Observe Measurer: Does/how does resource differentiate observers and proxies, does it address the issue of judgment?

1. Does resource differentiate observers and proxies? Y/N

If Yes, describe how:

1. Does resource discuss judgment in observer reporting? Y/N

If Yes, describe how:
